# Can teaching serious illness communication skills foster multidimensional empathy? A mixed-methods study

**DOI:** 10.1186/s12909-023-04010-z

**Published:** 2023-01-11

**Authors:** Jacqueline K. Yuen, Christopher See, Johnny T. K. Cheung, Chor Ming Lum, Jenny SW Lee, Wai Tat Wong

**Affiliations:** 1 Department of Medicine, The University of Hong Kong, Queen Mary Hospital, 4/F Professorial Block, 102 Pokfulam Road, Pokfulam, Hong Kong SAR, China; 2 School of Biomedical Sciences, The Chinese University of Hong Kong, Hong Kong SAR, China; 3 Faculty of Medicine, The Chinese University of Hong Kong, Hong Kong SAR, China; 4 Department of Medical and Geriatrics, Shatin Hospital, Hong Kong SAR, China; 5 Department of Medicine, Alice Ho Miu Ling Nethersole Hospital, Hong Kong SAR, China; 6 Department of Anaesthesia & Intensive Care, The Chinese University of Hong Kong, Hong Kong SAR, China

**Keywords:** Communication skill training, Empathy, Serious illness communication, Medical students, Mixed methods research

## Abstract

**Background:**

To investigate the impact of a serious illness communication skills training course on medical students’ attitudes regarding clinical empathy, self-efficacy in empathic communication, and learning on different dimensions of empathy.

**Methods:**

A mixed-methods design was used. A blended learning Serious Illness Communication Skills Training (SI-CST) course was delivered to sixth-year medical students. Students (*n=*185) completed questionnaires with the 20-item Jefferson Scale of Empathy (JSE) and self-rated preparedness level for five empathic communication tasks at baseline (T1), six weeks (T2), and three-to-six months post-training (T3). Written reflections on key lessons learned were analyzed using inductive thematic analysis.

**Results:**

Total JSE scores significantly improved from T1 to T2 (111.4 vs 113.9, *P*=.01) and from T1 to T3 (111.4 vs 113.9, *P=*.01). There was an increase in *Standing in Patient’s Shoes* subscale of the JSE with an effect size of 0.56 whereas the *Perspective-Taking* and *Compassionate Care* subscales showed no significant changes. Self-rated preparedness for all five empathic communication tasks significantly improved from T1 to T2 (*P* ≤ .001) and from T1 to T3 (*P* ≤ .001) with large effect sizes (1.09-1.41). Four key themes emerged from the qualitative analysis. They were appreciating the important role of empathy in clinical care (moral empathy), learning skills in detecting and understanding patient emotions (cognitive empathy), learning skills in responding to emotion with empathy (behavioral empathy), and appreciating that communication skills can be improved with continual practice and self-reflection.

**Conclusions:**

Our results revealed that SI-CST improved medical students’ empathic attitudes and self-efficacy in empathic communication. Qualitative results found learning on the cognitive, behavioral and moral dimensions of empathy.

**Supplementary Information:**

The online version contains supplementary material available at 10.1186/s12909-023-04010-z.

## Background

Empathy provides an important foundation for a positive doctor-patient relationship and contributes to improved patient outcomes [1–4]. Medical schools and medical associations have identified the cultivation of clinical empathy an important goal in medical training [5, 6]. Although there is no consensus on the definition of clinical empathy, it is generally understood as a multidimensional concept [7, 8]. Morse et al describes the four dimensions of clinical empathy as 1) cognitive, the intellectual ability to identify and understand the patients’ emotions and perspectives; 2) emotive, the ability to subjectively experience and share in the patients’ emotions; 3) behavioral, the ability to convey understanding of patient’s emotions and perspectives, and 4) moral, the internal altruistic force that motivates the practice of empathy [9]. Among these dimensions, cognitive empathy and behavioral empathy, are considered core components in most conceptualizations of clinical empathy [10, 11].

Communication skills training (CST) is one of the most commonly used educational interventions to cultivate empathy in medical students [12, 13] CST is thought to have most effect on cognitive and behavioral empathy because these are skills rather than personality traits, and thus more amenable to training [13–15]. Researchers in health communication, however, have argued that for healthcare professionals to adopt new communication behaviors, training should not only address skill-based outcomes and knowledge acquisition, but also affective outcomes such as attitudes and motivational factors [16]. This view on CST is consistent with Bandura’s social learning theory that identified self-efficacy and outcome expectancy beliefs to be key factors to change an individual’s behavior [17].

Existing CST interventions for empathy training have rarely assessed the impact on learners’ attitudes, self-efficacy, and motivation to practice clinical empathy and how these relate to learning on different dimensions of empathy. We postulate that effective CST that addresses skills-based and affective outcomes can impact on multiple dimensions of empathy. The aim of this study was to evaluate the impact of a Serious Illness Communication Skills Training (SI-CST) intervention on medical students’ attitudes regarding empathy in clinical care, self-efficacy in empathic communication, and learning on different dimensions of empathy. We employed a mixed-methods triangulation design including pre/post-training self-reported measures and qualitative analysis of students’ written reflections to understand the effects of SI-CST on clinical empathy.

## Methods

### Participants

Participants were final year medical students in the six-year program at the Faculty of Medicine, The Chinese University of Hong Kong. SI-CST was delivered as a mandatory course within a newly reformed communication skills curriculum. All students who enrolled in SI-CST were invited to participate in the study. This study was approved by the Survey and Behavioral Research Ethics Committee at The Chinese University of Hong Kong.

### Serious illness communication skills training (SI-CST)

**Fig. 1:**
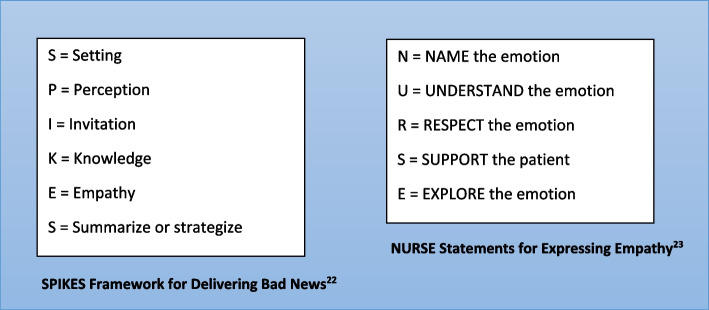
SPIKES framework for delivering bad news and NURSE statements for responding to emotion taught in SI-CST

SI-CST employed evidence-based approaches for CST including deliberate practice [18, 19], experiential learning [20], and self-reflections [21] delivered through a blended learning format. The course spanned a one-week period during the Internal Medicine clerkship. It consisted of both asynchronous online modules and a face-to-face tutorial totaling approximately five learning hours as follows:

1. Pre-tutorial online modules (2 hours): The first modules contained didactics on the SPIKES framework [22] for breaking bad news and skills in empathic communication. The latter involved skills in identifying and exploring emotional cues, and using nonverbal and verbal ways to respond to emotion including NURSE statements [23] (Fig 1). The following modules consisted of video-based exercises for practice in identifying communication skills covered in the didactics. The final module was a reflective writing exercise where students wrote about a prior serious illness communication encounter they observed and reflected on the effectiveness of the clinician’s communication skills based on the newly learned communication frameworks and skills.
2. Small group tutorial (2 hours): This face-to-face tutorial consisted of 6 students and led by 1-2 facilitators who are physician specialists in geriatrics, palliative care, or critical care medicine experienced in serious illness communication. First, the students shared and discussed their written reflections and then reviewed the communication frameworks and skills. The majority of the session was dedicated to skills practice through experiential role-play. During each role-play, one student enacted the role of the “patient” or “family member,” another the “doctor” while the rest were observers. After the role-play, the “doctor” self-reflected on the encounter, listened to the reflections of the “patient” or “family member,” as well as received feedback from peer observers and facilitators. In the last part, the facilitators introduced the concept of deliberate practice and encouraged use of deliberate practice and self-reflection to continue to improve communication skills in their day-to-day clinical practice, particularly in challenging communication encounters.
3. Post-tutorial online modules (1 hour): These modules included interactive video-based exercises designed to consolidate the knowledge and skills learned in the course. In the first module, students reviewed video clips of clinicians with varying skill levels in serious illness encounters and provided a written feedback to the clinician. The students were then shown an expert’s detailed feedback to the clinician. In the second module, the students were tasked with writing an appropriate response to challenging communication scenarios shown on video, and then shown video clips of expert-level physicians responding to these scenarios and the rationale for their responses. At the end of the modules, students were asked to reflect on their learning and write down two key learning points from the exercises.

The students’ completion of the online modules was tracked using the online learning platform and attendance at the small group tutorial was recorded by the facilitators. Students were required to complete all components of SI-CST to pass the course.

### Data collection

Students completed a self-administered questionnaire at three time points: immediately prior to the course (T1), two weeks post-training (T2) and three-to-six months post-training (T3). Data collection was conducted between June 2018 and June 2019. A unique participant identifier linked questionnaires completed by the same individual across the three time points. The questionnaires included demographic information, intended choice of specialty, and the following measures:

*JSE*. The Jefferson Scale of Empathy – Student version (JSE-S) is a validated instrument designed to measure medical students’ orientation or attitudes toward physician empathy in patient care. The instrument has 20 items rated on a 7-point Likert-type scale (1 = strongly disagree, 7 = strongly agree). The possible scores range is 20 to 140, with a higher score indicating a higher empathic orientation. Factor analysis identified three factors in the scale: *Perspective Taking* (10 items), *Compassionate Care* (8 items)*,* and *Standing in the Patient’s Shoes* (2 items) [11]. The scale was found to have high internal consistency (Cronbach’s alpha coefficient 0.80) [24] and substantial discrimination power [25].

*Self-rated preparedness level for empathic communication.* To assess students’ self-efficacy in empathic communication, the students rated their preparedness level for five empathic communication tasks selected by the study investigators based on the course objectives and literature review. The items were rated on a *5*-point Likert scale (1=not at all prepared, 5=extremely prepared). Task-specific self-efficacy ratings of communication behaviors have been found useful for assessment of learners’ communication training needs and training outcomes [16].

### Statistical analysis

We conducted paired sample *t*-test to compare JSE and empathic communication preparedness scores between baseline and six weeks post-training (T1 vs T2) to assess short-term changes, between baseline and three-to-six months post-training (T1 vs T3) to assess medium-term changes, and between six weeks and three-to-six months post-training (T2 vs T3). Cohen’s d effect sizes were calculated to identify significant differences between scores at T1 and T3.

Given the paired sample t-test was performed for the three pairwise comparisons, we used the Bonferroni-adjusted *P* value of 0.017 as the significance level to reduce the risk of type I errors [26]. The data were analyzed using IBM SPSS Statistics 25 for Windows.

### Qualitative analysis

At the completion of the course, the students were asked to write a response to the open-ended question, “What are the two most important things I learned from this course?”

The investigators used inductive thematic analysis of the written responses to explore how the course influenced student learning of clinical empathy. First, two investigators individually coded all the written responses using NVivo qualitative data analysis software. The investigators then discussed their codes and reached consensus on the coding scheme and the themes that emerged. The focus of the analysis was on the portions relevant to clinical empathy. The process continued iteratively until thematic saturation was reached. The qualitative findings were then used to triangulate with the results from the quantitative measures.

## Results

### Participants

Out of 185 recruited students, 100% completed all components of SI-CST. All 185 students completed questionnaires at T1, 142 (76.7%) completed questionnaires at T2, and 145 (78.4%) at T3. Ninety-eight students (53.0%) completed questionnaires at all 3 time points. Table 1 presents the characteristics of the participants. Mean age was 23.4 and 54.1% were female. The most common intended specialty choices were internal medicine (28.1%) followed by surgery (25.4%).

**Table 1:** Characteristics of sixth-year medical students participating in Serious-Illness Communication Skills Training (*n=*185)

Characteristic	Mean (SD)	N (%)
Age	23.4 (1.6)	
Gender		
Male		85 (45.9)
Female		100 (54.1)
Intended specialty		
Internal Medicine		52 (28.1)
Surgery		47 (25.4)
Obstetrics/Gynecology		12 (6.5)
Pediatrics		10 (5.4)
Family Medicine		8 (4.3)
Psychiatry		8 (4.3)
Emergency Medicine		7 (3.8)
Ophthalmology		5 (2.7)
Radiology		3 (1.6)
Pathology		2 (1.1)
Anesthesiology		1 (0.5)
Undecided		30 (16.2)

*SD* Standard deviation

### Changes in Jefferson Scale of Empathy Scores

Table 2 shows the mean JSE scores of the 98 students who completed all questionnaires at T1, T2, and T3. Total JSE scores were significantly higher at T2 and T3 than at T1. There was no significant change in the total JSE scores from T2 to T3. The effect size of the increase from T1 to T3 was 0.25.

**Table 2:** Changes in JSE scores at baseline (T1), six-week follow-up (T2), and three-to-six month follow up (T3)

		T1 Mean (SD)	T2 Mean (SD)	T3 Mean (SD)	Effect size^a^	Differences^b^		
	N					T1 vs T2	T2 vs T3	T1 vs T3
JSE Total	98	111.38 (9.06)	113.87 (9.34)	113.92 (11.17)	0.25	T1<T2*	T2=T3	T1<T3*
Perspective taking	98	57.9 (4.99)	58.29 (5.26)	58.36 (6.06)	0.08	T1=T2	T2=T3	T1=T3
Compassionate Care	98	44.66 (4.76)	45.38 (4.15)	45.46 (5.1)	0.16	T1=T2	T2=T3	T1=T3
Standing in Patients’ Shoes	98	8.81 (2.63)	10.19 (1.86)	10.1 (1.91)	0.56	T1<T2**	T2=T3	T1<T3**

*SD* Standard deviation, *JSE* Jefferson scale of empathy

^*^Denotes *P* <0.017

^**^Denotes *P* < 0.001

^a^Cohen’s d effect sizes were calculated to identify significant differences in scores between T1 and T3

^b^Paired t-test

The subscale scores for *Standing in the Patient’s Shoes* were significantly higher at T2 and T3 compared to T1 and remained unchanged from T2 to T3. The effect size of the increase from T1 to T3 was 0.56. For the subscales *Perspective Taking* and *Compassionate Care*, there was a trend showing increased scores from T1 to T3 although not statistically significant.

### Changes in self-rated preparedness for empathic communication

Students’ self-rated preparedness for all five empathic communication tasks significantly improved from T1 to T2, T2 to T3, and T1 to T3 (*p<0.001*) (Table 3). All five empathic communication tasks showed large effect sizes from T1 to T3 (range 1.09 to 1.41). The greatest increase was for *Respond to a patient or family member’s emotions* (effect size 1.41), followed by *Use verbal expressions of empathy in serious illness conversations* (effect size 1.27), and *Use nonverbal communication in serious illness conversations* (effect size 1.21).

**Table 3:** Changes in preparedness level for empathic communication tasks at baseline (T1), six-week follow-up (T2), and three-to-six month follow up (T3)

		T1 Mean (SD)	T2 Mean (SD)	T3 Mean (SD)	Effect size^a^	Differences^b^		
	N					T1 vs T2	T2 vs T3	T1 vs T3
Discuss bad news with a patient/family member about serious illness	98	2.64 (0.72)	3.08 (0.55)	3.41 (0.61)	1.15	T1<T2**	T2<T3**	T1<T3**
Respond to a patient/family member’s emotions	98	2.47 (0.69)	3.11 (0.55)	3.42 (0.66)	1.41	T1<T2**	T2<T3**	T1<T3**
Use nonverbal communication in serious illness conversations	98	2.86 (0.70)	3.42 (0.63)	3.68 (0.67)	1.21	T1<T2**	T2<T3**	T1<T3**
Use verbal expressions of empathy in serious illness conversations	98	2.78 (0.68)	3.31 (0.62)	3.61 (0.64)	1.27	T1<T2**	T2<T3**	T1<T3**
Elicit a patient/family’s needs or concerns	98	2.87 (0.65)	3.24 (0.69)	3.57 (0.64)	1.09	T1<T2**	T2<T3**	T1<T3**

*SD* Standard deviation

^**^Denotes *P* <0.001

^a^Cohen’s d effect sizes were calculated to identify significant differences in scores between T1 and T3

^b^Paired t-test

### Reflections on most important lessons from serious illness CST

One hundred sixty-nine students (91.4%) submitted written responses on the most important lessons learned. We identified four major themes related to clinical empathy as follows:

1. Appreciating the important role of empathy in clinical care
2. Learning skills in detecting and exploring emotional cues
3. Learning skills in responding to emotion with empathy
4. Appreciating that empathic communication skills can be improved with training and practice

### Appreciating the important role of empathy in clinical care

A key dimension of our teaching impact expressed by students was enhancing appreciation of the importance of understanding patients’ and their relatives’ needs and concerns when providing care to them as future physicians:

“Breaking bad news is an art, sometimes as medical students, we focus on the knowledge and what we know. But to patients, we need to address their concerns and their wishes and understand what they think is best for themselves or their relative.”

The impact on attitudes towards empathy in clinical care was further illustrated by appreciation of the complex potential benefits of empathic exploration with the patient. Our students perceived benefit to both informational and emotional support for patients and the further dimension of the therapeutic role of empathy and its facilitation of patient management:

“Exploring the patient/family’s needs is important to truly support the patient/family. That helps us to provide tailor-made information and support that is really useful and appropriate. At the same time, the patient/family will be emotionally supported if their concerns are responded to.

“Empathic responses are important and vital for calming the patient to allow room to break bad news and discuss management cognitively.”

Students also described changes in their attitudes regarding the role of empathy in establishing a good doctor-patient relationship.

“Expressing empathy is key to good communication and exchange of information between the doctor and the patient and/or family. It is not time-consuming or labor intensive to provide empathic responses and once empathy is channeled from the doctor to the patient and family, there is an immense improvement in the atmosphere of the conversation. It also helps with strengthening the doctor-patient relationship.”

These comments reflect the student’s perceived impact of SI-CST on shaping their attitudes regarding the role of empathy in clinical care. These attitudes foster moral empathy, or their altruistic motivation to practice empathy.

### Learning skills in detecting and exploring emotional cues

Another goal of the course was to enhance skills in perspective-taking, such as active listening and detecting emotional cues, which could represent learning on cognitive empathy.

A number of students described learning such skills to improve their ability to detect emotional cues and understand the perspectives of patients and family members.

“I learnt to be observant and understand that emotions can be embedded in the patient's question/ nonverbal responses. These responses need to be addressed with empathetic responses instead of rational answers.”

“Listening to the patient instead of focusing on our own agenda in a family meeting”

Students also described how the course gave them experiential learning opportunities to explore emotional cues which enable them to understand the patients and family’s feelings and perspectives.

“Through this course and the online materials, I was able to experience the importance of exploring the family and the patient's feelings and wishes before continuing the conversation. This reminds me that it is not just the simple act of being aware and empathetic, it is more about getting to know the entire situation and any hidden concerns.”

### Learning skills in responding to emotion with empathy

Students also found the course useful in learning skills in expressing empathy including both non-verbal and verbal expressions. The students described the learning undertaken through the course in context of their previous uncertainties surrounding empathic responses, allowing us to understand aspects of the value added and perspectives gained by their participation in the intervention:

“The NURSE method of providing empathetic responses is a good framework, because often times we may want to provide empathetic responses but we are not sure how to express ourselves. This gives us a framework of what we can say during those times.”

“Other than empathic responses, silence is a very powerful tool to show that we are present with the family to endure the difficult times and provide as much support as possible.”

Some students also highlighted learning skills in communicating one’s understanding of another’s emotions which represent gains in behavioral empathy.

“Empathy is not achieved until I properly convey my understanding to the counterpart.”

### Appreciating that empathic communication skills can be improved with training and practice

In their reflections, students shared their appreciation that empathic communication is a learnable skill. This indicates an important attitudinal change encouraged by our intervention which is in keeping with principles of lifelong learning and a growth mindset.

“Expression of empathy is a skill that can be learnt.”

“The correct use of empathy depends a lot on the ability to pick up emotional cues, and it is always something we need more training for.”

Students also expressed behavioral intentions to engage in continual practice for further improvement. This gave some insight to the future potential value and possible clinical impact of the intervention. Such comments were sometimes associated with self-reflective thoughts which further support this forward-looking dimension of the attitudinal impact of the intervention.

“Feedback from the post-course questions and tutors during the tutorials are useful to determining my own weaknesses, such that I will be more alert and focused on those during future practices.”

“I have come to know the importance of reflecting and improving on my own communication skills after each difficult consultation during my journey of becoming and being a doctor in order to conduct better communication with patients and relatives.”

These comments related to the students’ belief that their empathic communication skills can be improved represents their self-efficacy and motivation to practice empathic communication in the future.

## Discussion

This study found that the one-week SI-CST led to improvements in final year medical students’ attitudes regarding empathy in clinical care and self-efficacy in empathic communication. The improved attitudes were sustained over a period of three-to-six months while their self-efficacy continued to increase during this period. Qualitative analysis supported the quantitative outcomes in that students learned skills in identifying and understanding emotional cues (cognitive empathy), skills in responding to emotion (behavioral empathy), and developed improved attitudes regarding the important role of empathy in clinical care (moral empathy). They also expressed motivation to practice empathic communication skills in the future, fueled by the belief they are learnable skills that can be improved.

**Fig. 2:**
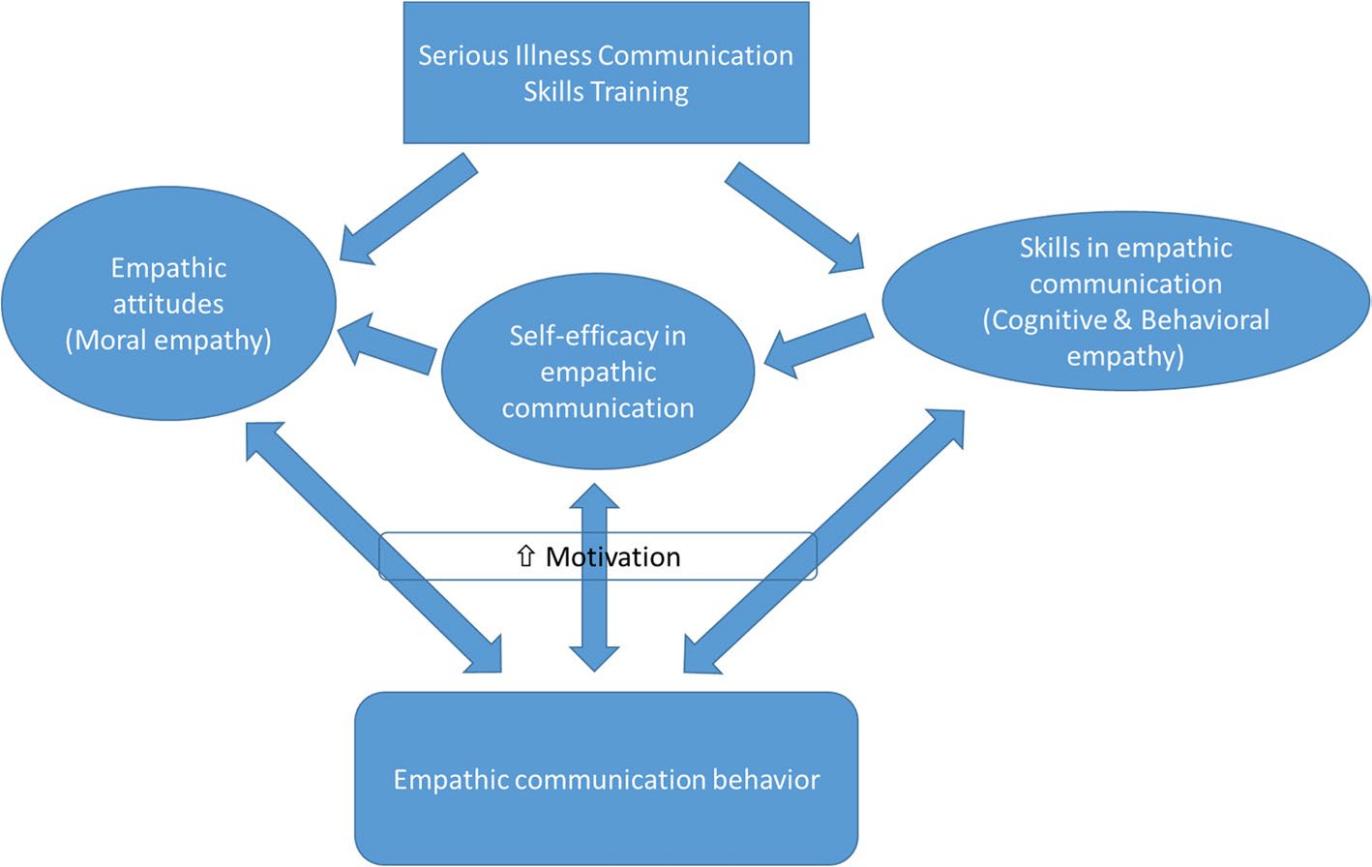
Model on effects of SI-CST on interplay of skills, attitudes, self-efficacy and learning on dimensions of empathy

We observed the largest effect of SI-CST was on increasing students’ self-efficacy in empathic communication and it continued to augment with time post training. We hypothesize that the newly acquired skills, coupled with the belief that their communication skills can improve with practice and a positive attitude regarding the role of empathy in patient care, contributed to this effect. Figure 2 provides a model for the potential effects of SI-CST. Improved self-efficacy may have motivated the students to put their newly learned skills to use in patient interactions. This is supported with Bandura’s theory whereby the combination of outcome expectancy (beliefs about the anticipated consequences of a behavior) and self-efficacy (beliefs on one’s ability to perform a behavior successfully) can motivate an individual to persist in performing the behavior, even during difficult situations [17]. By practicing the communication skills acquired through SI-CST, the students developed increasing mastery of the skills with time, which may explain the further gains in self-efficacy that we observed.

With regards to changes in JSE, a measure of attitudes regarding empathy in clinical care, a moderate effect size was observed in the increase in *Standing in the Patient’s Shoes* subscale scores. This subscale contains two items related to the *difficulty* in seeing things from the patients’ perspectives (namely *“Because people are different, it is difficult to see things from patients’ perspectives”* and *“It is difficult for a physician to view things from patients’ perspectives”*). This subscale is conceptually related to self-efficacy in perspective taking. Given the increase in self-efficacy in empathic communication we observed, it follows that students’ perceived difficulty in seeing things from a patients’ perspective was lessened after SI-CST. Therefore, improving self-efficacy in empathic communication can itself exert a positive influence on attitudes regarding empathy in clinical care (Fig 2).

On the other hand, we found a modest but not statistically significant effect on the *Perspective Taking* and *Compassionate Care* subscales. The *Perspective Taking* subscale relates to attitudes regarding the role of cognitive empathy (e.g. *“Physicians should try to think like their patients in order to render better care”*) [11]. Although some students reported appreciating the importance of perspective taking in their written responses, the incremental change may not have been large enough to be reflected in changes in the *Perspective Taking* subscale. The *Compassionate Care* subscale represents attitudes regarding the role of emotive empathy (e.g. *“Patients’ illnesses can be cured only by medical or surgical treatment; therefore, physicians’ emotional ties with their patients do not have a significant influence in medical or surgical treatment”*). The lack of significant effect on the *Compassionate Care* subscale is consistent with lack of reported learning on emotive empathy in their written responses.

Thus, the impact of SI-CST appears strongest on the cognitive, behavioral and moral dimensions of empathy, and have little or no effect on emotive empathy. Emotive empathy, which Morse defines as “the ability to subjectively experience and share in another’s psychological state or intrinsic feelings,” is more akin to sympathy [9]. Overemphasis on emotive empathy may be problematic for physicians to maintain objectivity and avoid emotional burnout and may not be a desired effect in empathy training [7, 10].

Prior CST intervention studies that evaluated effects on empathy in medical students using the JSE have shown mixed results where some studies found no improvement [27], while others have observed partial or significant improvements [28–30]. While these CST interventions have in common the use of role-play, there is heterogeneity in their design including the year of target participants, number of training hours, use of simulated patients, and the experience and training of facilitators. Many of these studies did not explore the mechanisms behind the effects they found. The current study contributes to this understanding by demonstrating the impact on SI-CST on the interplay between self-efficacy and attitudes and how these are related to learning on different dimensions of empathy.

We believe certain elements of SI-CST made it conducive to empathy training and can be adopted by training programs in other medical schools. The first is incorporating experiential learning in serious illness communication through role-play. Students gained experience in challenging emotional situations such as breaking bad news about a life-limiting illness in a safe setting. Such experiences made them more attuned to psychosocial dimensions of care which can foster empathy [31]. Second, in lieu of hired actors, the course enabled some students to act in the role of seriously ill patients or family members. These students were given a detailed description of the “character” and additional time to prepare before the role-play. The benefit of this approach was demonstrated in a New Zealand study that employed drama training in “how to act-in-role” for medical students. Trained students had significantly higher JSE scores and better performance in clinical communication examinations [32]. The third important element was the selection of facilitators who are clinicians experienced in serious illness communication. Through sharing their own experiences in serious illness encounters, the facilitators served as role models and their feedback felt credible to the students. This sentiment was reflected in many of the students’ comments in the course evaluation. Finally, the emphasis on deliberate practice and self-reflection provided tools for learners to continue to engage in communication skills practice independently after SI-CST. Some ways in which SI-CST can be further enhanced in the future as suggested in the student evaluations are to provide additional role-play sessions for skills practice and more demonstration videos to model good communication behaviors in different scenarios.

This study has some limitations. First, this study did not use a randomized, controlled design given the compulsory nature of the course. Nonetheless, we did not identify significant differences in the pre- and post-training scores of students who completed the course in Term 1 vs Term 2 to suggest presence of cofounding factors related to the timing of the course (Supplementary file 1). Second, the response rate of students who completed all questionnaires was 53%. Despite this response rate, we believe the likelihood of selection bias is low given there were no significant differences in the JSE and preparedness scores between students who responded at all three time points and those at only two time points. Third, since this study used self-report measures of empathy, we cannot determine whether students demonstrated empathic behaviors in patient interactions post training. Nonetheless, prior studies have shown that self-report empathy measures were significantly associated with evaluators’ ratings of empathic behaviors [32, 33] and with patients’ evaluation of clinician empathy [34].

## Conclusion

Despite studies that suggest a declining trend in empathy as medical students progress from pre-clinical years to the clinical years [35–37], our study found that SI-CST improved attitudes and self-efficacy in empathic communication among final-year medical students that were sustained at least in the medium-term. The training led to learning on the cognitive, behavioral and moral dimensions of empathy. Future research is needed to assess whether SI-CST can result in observable improvement in communication behaviors.

## Supplementary Information

**Additional file 1:** **Supplementary file 1.** Comparison of JSE scores and preparedness level for empathic communication tasks between students completing the Serious Illness Communication Skills Training in Term 1 versus Term 2.

## Data Availability

The datasets used and/or analyzed during the current study are available from the corresponding author on reasonable request.

## Abbreviations

CST: communication skill training
JSE-S: Jefferson Scale of Empathy – Student Version
SI-CST: Serious illness Communication Skills Training
T1: Baseline/Immediately prior to training
T2: Two weeks post-training
T3: Three-to-six months post-training
