# Supplementary material for: Can teaching serious illness communication skills foster multidimensional empathy? A mixed-methods study
Source: BMC Med Educ. 2023 Jan 11;23:20. doi: 10.1186/s12909-023-04010-z (PMC9835381; doi:10.1186/s12909-023-04010-z)
Supplement: Supplementary file 1 — Additional file 1: Supplementary file 1. Comparison of JSE scores and preparedness level for empathic communication tasks between students completing the Serious Illness Communication Skills Training in Term 1 versus Term 2. [file 12909_2023_4010_MOESM1_ESM.docx]

|  | Term 1 | Term 2 |  | Term 1 | Term 2 |  |
| --- | --- | --- | --- | --- | --- | --- |
|  | T1  Mean (SD) (n=88) | T1  Mean (SD)  (n=97) | P value^a^ | T3  Mean (SD) (n=76) | T3  Mean (SD)  (n=69) | P value^a^ |
| **JSE** |  |  |  |  |  |  |
| Total | 110.20 (9.37) | 110.31 (8.84) | 0.93 | 112.63 (12.25) | 113.10 (9.51) | 0.80 |
| Perspective taking | 57.12 (4.91) | 57.11 (5.17) | 0.99 | 57.1 (6.80) | 57.19 (5.49) | 0.97 |
| Compassionate care | 44.17 (4.93) | 44.54 (4.10) | 0.58 | 45.32 (5.22) | 45.91 (3.80) | 0.44 |
| Standing in Patients’ shoes | 8.91 (2.51) | 8.66 (2.41) | 0.50 | 10.16 (1.47) | 10.00 (1.37) | 0.51 |
|  |  |  |  |  |  |  |
| **Preparedness for empathic communication tasks** |  |  |  |  |  |  |
| Discuss bad news with a patient/family about serious illness | 2.57 (0.80) | 2.71 (0.56) | 0.18 | 3.32 (0.66) | 3.46 (0.61) | 0.19 |
| Respond to patient/family emotions | 2.47 (0.76) | 2.49 (0.65) | 0.78 | 3.42 (0.66) | 3.41 (0.60) | 0.83 |
| Use nonverbal communication in serious illness conversations | 2.80 (0.78) | 2.79 (0.65) | 0.97 | 3.61 (0.76) | 3.63 (0.67) | 0.86 |
| Use verbal expressions of empathy in serious illness conversations | 2.73 (0.71) | 2.78 (0.64) | 0.60 | 3.57 (0.70) | 3.68 (0.68) | 0.34 |
| Elicit patient/family concerns or needs in the setting of serious illness | 2.77 (0.80) | 2.79 (0.61) | 0.84 | 3.51 (0.68) | 3.54 (0.56) | 0.78 |

**Supplementary file 1.** Comparison of JSE scores and preparedness level for empathic communication tasks between students completing the Serious Illness Communication Skills Training in Term 1 versus Term 2.

*SD* standard deviation, *JSE* Jefferson Scale of Empathy ^a^Independent t-test
